# Diagnostics of Ovarian Tumors in Postmenopausal Patients

**DOI:** 10.3390/diagnostics12112619

**Published:** 2022-10-28

**Authors:** Chingis Mustafin, Sergey Vesnin, Arran Turnbull, Michael Dixon, Alexey Goltsov, Igor Goryanin

**Affiliations:** 1 Medical Microwave Radiometry (MMWR) Ltd., Edinburgh EH10 5LZ, UK; 2 Edinburgh Breast Unit, University of Edinburgh, Edinburgh EH8 9YL, UK; 3 Institute for Artificial Intelligence, Russian Technological University (MIREA), 119454 Moscow, Russia; 4 School of Informatics, University of Edinburgh, Edinburgh EH8 9YL, UK; 5 Okinawa Institute Science and Technology Graduate Universality, Okinawa 904-0495, Japan

**Keywords:** post-menopause, tumor marker, ovarian tumors, ovarian lesions, medical microwave radiometry

## Abstract

Early diagnosis of ovarian cancer remains an urgent issue owing to the continuing trend towards increasing incidence along with only marginal improvements in mortality and 5-year survival rates. Furthermore, there is a lack of a clear formulation of the concept of pathogenesis. The diagnostic values of tumor markers, their potential advantages and disadvantages, and their combination with radiation imaging methods and transvaginal sonography are discussed. More advanced imaging techniques, such as computed tomography and magnetic resonance imaging have proven too expensive for widespread use. According to the World Health Organization, more than half of the world’s population does not have access to diagnostic imaging. Consequently, there is high demand for a low-cost, reliable, and safe imaging system for detecting and monitoring cancer. Currently, there is no clear algorithm available for examining and accurately diagnosing patients with postmenopausal ovarian tumors; moreover, reliable criteria allowing dynamic observation and for determining surgical access and optimal surgical intervention measures in postmenopausal patients are lacking. Medical microwave radiometry shows promising results yielding an accuracy of 90%.

## 1. Introduction

The diagnosis and treatment of ovarian neoplasms remain crucial in gynecology [1]. The relevance of this issue is due to the widespread prevalence of ovarian tumors and tumor-like formations as well as the continuing upward trend of ovarian cancer incidence along with only marginal improvements in mortality and 5-year survival rates [2]. In several countries, ovarian cancer ranks eighth (>4% frequency) among all malignant neoplasms in women of all age groups [3,4]. Ovarian neoplasms account for up to 25% of all tumors of the female genital organs, and worldwide incidence has been shown to peak in the early postmenopausal period around the ages of 55–64, with a median age at diagnosis of 63. Approximately 25 million women worldwide pass through menopause every year. By the year 2030, there will be 1.2 billion menopausal and postmenopausal women, with 47 million new additions each year [5]. The average age of menopausal women ranges from 49 to 51 years, and women live almost one-third of their lives in a condition of estrogen deficiency [6]. Moreover, benign ovarian tumors are prevalent in 2.5–18% of postmenopausal women [7].

Screening, which allows for the diagnosis of a malignant ovarian tumor at the early asymptomatic stage of development, is the most important and optimal way to reduce mortality. Screening for ovarian cancer is not recommended for women in the general population nor women without risk factors (low-risk group). There is currently no clear algorithm available for examining ovarian tumors in women; furthermore, no reliable criteria are available to select the optimal management strategy, such as dynamic observation or surgical intervention [8], for each patient. Therefore, obstetricians and gynecologists need to urgently explore new research methods that would allow reliable differential diagnosis of tumors and tumor-like formations in the ovary at the prehospital stage.

Existing imaging systems based on ionization radiation have limited permissible exposure dosages and cannot be used frequently. In recent years, much effort has been devoted to finding a reliable cancer diagnostic tool using non-ionization radiation. For example, in an experimental study by Alibakhshikenari and colleagues [9], a standard biomedical breast model that mimics a real human breast in terms of dielectric and optical properties was used to demonstrate the viability of a novel antenna for a microwave breast imaging system. Currently, passive microwave radiometry (MWR), which allows for the visualization of natural electromagnetic radiation from human organs in the range of 3.4–4.2 GHz, is garnering interest from researchers.

## 2. Materials and Methods

In this study, we reviewed different methods currently available for ovarian cancer diagnostics. Existing methods for diagnosing ovarian cancer that are approved by the Society of Gynecologic Oncology and the American College of Obstetricians and Gynecologists include clinical examination with detailed history taking and assessment of symptoms, radiation imaging methods (transvaginal ultrasound, Doppler ultrasound, computed tomography (CT), magnetic resonance imaging (MRI), and positron emission tomography), genetic counselling for women at high risk of developing the disease, and the determination of biomarkers by various methods based on both protein expression and changes in genes. 

## 3. Results

Numerous studies have reported that systematic gynecological examination does not significantly affect the identification of ovarian formations [10,11]. Difficulties in the differential diagnosis of ovarian tumors are associated with limited clinical symptoms at the early stages of the disease [12,13]. Moreover, at present, no gold standard method for identifying malignant neoplasms at the borderline tumor stage and early stages of malignancy [14] is available worldwide. 

Echography of the pelvic organs in combination with the determination of serum carbohydrate antigen 125 (CA-125) levels is recognized as an ineffective means of screening for early-stage ovarian cancer [15]. Until 1981, the only biomarker used to diagnose ovarian cancer was the cancer embryonic antigen (CEA). CEA was first described in 1965 as a serum biomarker in mucinous colon cancer and as a marker in women with ovarian cancer [16,17]; however, in 1981, CA-125 was identified in ovarian, cervical, endometrial, gastrointestinal, and breast cancers [18]. CA-125 is presently the most common serum marker for ovarian tumors and is widely used for preoperative diagnostics, treatment evaluation, and monitoring of patients with ovarian cancer. Benjapibal and Neungton (2007) found that the detection of serum CA-125 alone has a certain degree of accuracy in differentiating malignant and benign ovarian tumors [19]; the specificity of the CA-125 test in benign ovarian tumors is 73.2%, whereas, in malignant tumors, it is approximately 99.3% [20,21].

While the CA-125 test is widely used to assess the effectiveness of treatment and prognosis of the disease, the use of several markers, particularly during the initial examination of patients with ovarian formations, would increase the likelihood of detecting a tumor [22]. Several studies have demonstrated the predictive values of the biomarkers cancer antigen 72-4, cancer antigen 15-3, CA-125, and vascular endothelial growth factor [23,24].

Currently, the human epididymis protein 4 (HE4) tumor marker is also commonly tested worldwide. HE4, a member of the protease inhibitor family, is formed in the epithelium of numerous tissues of the female genital tract (fallopian tubes, endometrium, and endocervix). The expression of the HE4 gene is markedly increased in ovarian cancer cells and owing to the low molecular weight (25 kDa) of the expressed protein, is abundantly found in the bloodstream. Many researchers have reported that patients at stages I–II of the disease show statistically significant increases in the level of HE4, which occurs at an even earlier stage than the significant rise of CA-125 [24,25,26]. According to a systematic review and meta-analysis by Lin et al. (2013), the sensitivity and specificity of HE4 in detecting epithelial malignant ovarian tumors were 74% and 87%, respectively, and 80% and 75%, respectively, for borderline tumors. Moreover, numerous studies have reported that in almost 50% of ovarian cancer cases with CA-125 values not exceeding the discrimination level, a significant increase was detected in HE4 [27]. Brown et al. (2008) emphasized this by showing that HE4 is increased in approximately 10% of cases of ovarian cancer with negative CA-125 indicators [28].

CA-125 in combination with carcinoembryonic antigen, alpha-fetoprotein, antigen 19-9, and Beta-HCG can improve diagnosis [29]. However, while CA-125 is associated with ovarian cancer, levels of this marker can also be elevated by the presence of lung, pancreatic, breast, and liver cancers, malignant tumors of the gastrointestinal tract, pelvic inflammatory disease in women, endometriosis, ovarian cysts, pancreatitis, and other pathologies.

The initial steps for creating an algorithm for the differential diagnosis of benign and malignant tumors were taken by Jacobs et al. (1990). To establish the most accurate risk factors (considering age, menopausal status, and level of the CA-125 marker), the authors developed a risk of malignancy index (RMI) with a sensitivity of 85% and specificity of 97%.

In 2010, Moore et al. proposed a new prognostic model, the risk of ovarian malignancy algorithm (ROMA) scale, which combines measurements of tumor markers CA-125 and HE4 [30,31]. The ROMA scale exhibited a sensitivity of 85.3% in patients with stage I and II ovarian cancer, compared with 64.7% in the case of the RMI scale (*p* < 0.0001). Furthermore, in 2011, Montagnana et al. found that the effectiveness of the ROMA scale is markedly higher in postmenopausal women than in premenopausal women [32]. Moreover, studies on the combination of markers HE4 and CA-125 have not shown better results than those of HE4 alone. In a meta-analysis by Dayyani et al. (2016), it was observed that the ROMA scale exhibited diagnostic advantages when compared with a study reporting only the levels of CA-125 and HE4 in patients with early ovarian cancer and postmenopausal women; in this group of patients, the effectiveness of the ROMA scale was higher than with the individual markers alone [33]. Furthermore, the RMI and ROMA methods have been shown to exhibit high sensitivity (83.3% and 75%, respectively) and specificity (95.3% and 100%, respectively) [34].

In 2016, a meta-analysis was carried out to identify an optimal predictive model for ovarian cancer, comparing the most common RMI assessment model, two International Ovarian Tumor Analysis (IOTA) models (simple ultrasound-based rules (simple rules) and Logistic Regression Model (LR2)), and the ROMA scale. It was revealed that the combination of IOTA simple rules and the subjective assessment by a doctor using ultrasound diagnostics gave the highest sensitivity and specificity (93% and 84%, respectively) in comparison with the RMI scale (75% and 92%, respectively) [34].

Needle biopsy and cytology can be used for ovarian cancer diagnostics [35]. Ultrasound mini-surgery can markedly increase the diagnosis of nosological types of volumetric pathological formations of the small pelvis, such as simple ovarian and peritoneal cysts, and helps to reduce the number of abdominal (unjustified) operations required [36]. However, the anatomical localization of the ovaries limits the widespread clinical use of cytology in ovarian tumors. Furthermore, invasive operations can cause the spread of tumor cells. These factors limit the use of mini-surgeries in the diagnosis of borderline tumors at the early stages of ovarian involvement. Therefore, the use of fine-needle biopsies of the ovaries in formations up to 50 mm in size holds critical significance.

Ultrasound has become the standard diagnostic method for evaluating epididymal tumors and is used as a first-line method in patients with suspicious isolated ovarian lesions [37]. This method mainly confirms the presence of a tumor, differentiates ovarian lesions from lesions of the uterus or fallopian tubes, and determines the internal structure of the tumor. Ultrasound is currently widely used in preoperative diagnosis, postoperative examination, and the long-term follow-up of patients with ovarian tumors. Using a combination of several modes of ultrasound imaging, the accuracy of differentiation between benign and malignant ovarian tumors is continuously improving. However, despite its widespread use, ultrasound is limited in its effectiveness at assessing the structure of tumors and their surrounding structures. To overcome this, ultrasound is being combined with dopplerometry to assess the characteristics of blood flow in ovarian formations and in conducting a more accurate differential diagnosis of tumors and tumor-like formations [38]. Several studies have reported that grey-scale ultrasound and color Doppler imaging cannot accurately determine the topical location and structure of the tumor at the preoperative stage [39].

Many national guidelines concerning the management of ovarian cancer currently advocate the RMI for the characterization of ovarian pathologies. However, other methods, such as subjective assessment and the IOTA simple rules and LR2 models, seem to be superior to the RMI [40].

To assess the accuracy of the IOTA LR2 scale for the diagnosis of ovarian cancer, a prospective single-center study of women with an ultrasound diagnosis of an adnexal tumor has been conducted. The subjects were examined by a single level II ultrasound operator who had received training in the systematic examination of ovarian tumors following the IOTA guidelines, and the likelihood of the adnexal lesion being malignant was calculated using the IOTA LR2 model. All women underwent surgery within 120 days of ultrasound examination, and the ultrasound findings were compared with the operative findings and final histological diagnosis. In total, 124 women were included in the final analysis. The mean age was 53.2 (range, 20–91) years, and 61/124 (49.2%) women were postmenopausal. Malignant lesions were found in 66/124 (53.2%) women on postoperative histological examination. The IOTA LR2 model had a sensitivity of 97.0% (95% confidence interval [CI], 89.5–99.6%) and a specificity of 69.0% (95% CI, 55.5–80.5%) [41,42,43,44,45,46,47,48,49,50,51,52,53].

As additional diagnostic methods, CT and MRI can be employed. These examinations alone are most often recommended for analyzing the prevalence of a malignant process as well as during observation in the postoperative period and the process of anticancer treatment. However, CT is ineffective in the primary detection and determination of tumor type in uterine appendages and assessment of local spread in the small pelvis. MRI can be used to determine the position, size, boundaries, components, and angiogenesis of tumors of the appendages; however, MRI, like CT, is not recommended for the primary assessment of postmenopausal ovarian cysts because of its low specificity and radiation exposure to the patient [13]. MRI should be used as an imaging technique if the ultrasound characteristics of an ovarian cyst are uncertain and inconclusive. 

Foci of elevated temperature (focal hyperthermia) in malignant neoplasms are thought to occur because of hypervascularization of the surrounding tissues and from the increased amount of thermal energy produced by cancer cells [54]. This can be visualized by MWR devices such as MWR2020 (formerly RTM-01-RES), which is certified and recommended for clinical use in several countries. The device visualizes electromagnetic radiation in the range of 3400–4200 MHz. The antenna diameter is 39 mm and provides a measurement depth of up to 40–50 mm [54,55,56,57]. Non-invasive measurements are conducted by registering electromagnetic radiation through the anterior abdominal wall (transabdominal) in the projection of the uterine appendages at nine symmetrical points on both sides. To establish the diagnosis, temperature indicators are considered only in the projection of the uterine appendages. Areas with a low temperature are visualized in blue (“cold”), and areas with an increased temperature are displayed in red and yellow (“warm”). 

In healthy women, the average internal temperature is 36.2 ± 0.1 °C, which is considered the healthy standard [58]. If the internal temperature differs from this by 0.5 °C or less, focal hyperthermia is not deemed to be present in the projection of the ovaries, ruling out a malignant neoplasm. Observation by a gynecologist is recommended with a follow-up study after 12 months, although a control is carried out after 6 months when menopausal hormone therapy (MHT) is being prescribed. When the internal temperature is 0.6–1.0 °C higher than the healthy standard in the projection of one or both ovaries, unexpressed focal hyperthermia is determined, which is a sign of a borderline tumor process preceding the development of a malignant tumor. A consultation with an oncologist is recommended, and MHT should not be used. When the internal temperature exceeds the healthy standard by 1.1 °C or more in the projection of one or both ovaries, pronounced focal hyperthermia is determined, indicating a high probability of a malignant process. A consultation with an oncologist and an examination in a specialized oncological institution is recommended. MHT is contraindicated.

**Figure 1:**
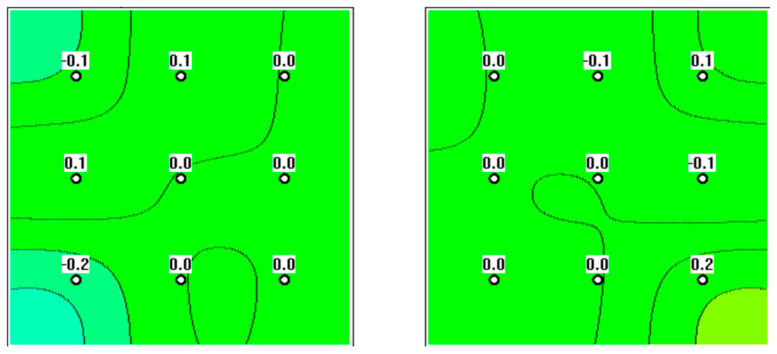
Microwave radiometry (MWR) assessment of a 65-year-old subject. The ovaries are normal, with no signs of focal hyperthermia.

**Figure 2:**
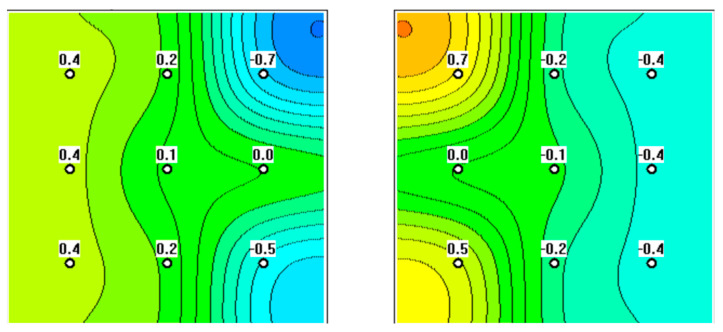
MWR performed on a 72-year-old patient. Signs of hyperthermia in the projection of the left ovary can be seen, putting the patient in the at-risk group.

**Figure 3:**
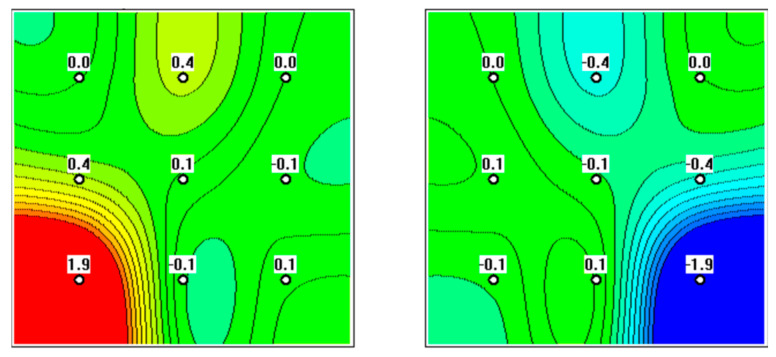
MWR performed on a 69-year-old patient. Focal hyperthermia in the projection of the right ovary is evident, putting the patient in the high-risk group.

**Figure 4:**
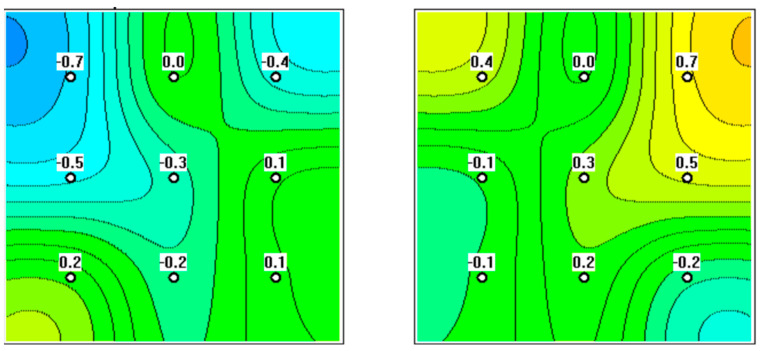
MWR was performed on a 59-year-old patient. Focal hyperthermia in the projection of the left ovary is evident, putting the patient in the high-risk group.

In a previous study, MWR was used to examine 119 women aged 49–73 years. The control group consisted of 54 healthy women. The accuracy of MWR was 90% [58]. Such studies demonstrate the utility of MWR in screening and diagnostics to determine the risk of developing ovarian cancer in postmenopausal women in the absence of symptoms, complaints, or clinical manifestations and to monitor MHT used in this age group. Furthermore, MWR could be used for screening of the iliac region when there are no preliminary data on the state of the ovaries and for targeted diagnostic radiometry of the iliac region when data on the state of the appendages are provided, obtained by other clinical and diagnostic methods (bimanual examination of the pelvic organs, ultrasound, etc.), which would allow for differential diagnosis of existing tumor formations in the ovaries in postmenopausal women. Thus, MWR provides high information content in a non-invasive, painless manner for the patient, which allows it to be performed repeatedly, is safe for any age group, and can be used for preventive examinations and dynamic monitoring of the state of the ovaries during MHT (Figure 1, Figure 2, Figure 3 and Figure 4) [58].

The examination of patients with ovarian formations includes a detailed study of the patient’s history, a two-handed vaginal examination, and additional general clinical research methods, such as the determination of tumor markers. However, small ovarian tumors are undetectable by manual palpation, and while ultrasound is a useful diagnostic tool, its successful application in ovarian formations depends on various factors, such as the class of equipment, the qualifications of the researcher, the visualization conditions of the pelvic organs, and the condition of the abdominal cavity concerning the body mass index and adhesions.

The determination of tumor markers, particularly CA-125, is critical in the diagnosis of ovarian tumors; however, this antigen is not strictly specific for ovarian formations, and its level can be increased by liver cirrhosis, acute pancreatitis, endometriosis, uterine myoma, and malignant tumors of the gastrointestinal tract and abdominal organs. The specific indications for surgery in postmenopausal patients with ovarian formations remain controversial because none of the existing diagnostic methods provide satisfactory sensitivity and specificity in practice.

Women experience menopause and conditions of estrogen deficiency for almost one-third of their lives. Post-menopause physiology leads to numerous disorders that contribute to an increase in chronic pathological conditions. The processes leading to such conditions could be caused by hormonal regulation (preservation of the receptor status, a high expression level of aggressive metabolites) or the induction of cellular proliferation via involutive atrophic processes, chronic inflammation, pronounced disorders of micropinocytosis, or induction of mutated stem cells.

Estrogen deficiency following menopause leads to irreversible changes in the organs and systems (disturbances in composition and metabolism, which can lead to the development of metabolic syndrome, cardiovascular diseases, osteoporosis, and osteopenia), which adversely affect the onset of ovarian neoplasms and hinder the diagnosis process. These features dictate the need for personalized approaches to diagnostics, management, and surgical tactics; with the high risk of anesthetic and surgical complications, which can severely deteriorate a woman’s health and quality of life, surgical intervention should be guided by clear indications. In addition, the need to surgically remove benign ovarian tumors of small sizes (up to 50 mm) in postmenopausal patients remains unclear considering that only a small percentage of these formations become malignancies.

## 4. Discussion

During 2001–2004, a study in the UK on 202,562 women aged 50–74 was conducted to assess the effectiveness of screening for ovarian cancer. The participants were randomized into three groups: the first group (50,625 women) took the CA-125 test annually, and if levels increased, the test was either repeated after 6 weeks or transvaginal sonography was performed; the second group (50,623 women) underwent transvaginal sonography annually, and if a malignancy was suspected, the CA-125 test was additionally performed; the third control group (101,314 women) only underwent observation, and examinations were carried if symptoms of the disease were present. All patients with diagnosed ovarian cancer received treatment at large hospitals of the national health system under the supervision of gynecological oncologists. The main criterion for effectiveness was mortality from ovarian and fallopian tube cancers.

With a median follow-up of 16 years, ovarian cancer was diagnosed in 2055 women, with a detection rate of 1% in all three groups. In the first group, where CA-125 was used as the main test, there was a higher rate of detection of the disease at stage I (by 47%) and a lower frequency at stage IV (by 25%) compared with the control group. In the group that underwent transvaginal sonography, the stage distribution of the identified patients did not differ significantly from the control group. However, earlier detection of the disease did not lead to either a decrease in mortality or a longer life expectancy of sick patients compared with the control group. A total of 1206 patients died, and the Kaplan–Meier survival curves for all three groups were the same; the number of patients who died was 0.6% in each group. This study showed that screening programs with CA-125 determination and transvaginal sonography are unable to reduce mortality from ovarian cancer, highlighting the need for a more accurate diagnostic method. The authors emphasized that it took more than 30 years from the beginning of the study of CA-125 testing and transvaginal sonography to obtain the results of the population-based randomized trial.

The early detection of malignant diseases of the ovaries and uterine appendages is especially important for women receiving MHT. This therapy is prescribed for peri- and postmenopausal women to treat symptoms associated with estrogen deficiency and to prevent the long-term consequences of hypoestrogenism. The positive effects of MHT (elimination of hot flashes, reduced risk of osteoporosis, etc.) are undeniable. At the same time, the problem of the possible influence of MHT on the risk of developing several malignant diseases, especially breast and genital cancers, has been widely discussed in recent scientific literature, although the metabolic effects of MHT components are still not sufficiently clear.

Despite the variety of diagnostic methods available, almost 65% of patients with ovarian cancer are admitted to specialized hospitals at advanced stages of the disease. Hence, the “active search” of patients at the preclinical phase of the development of the tumor process in women belonging to the “risk group” should be considered today as one of the most effective ways to prevent ovarian cancer. However, the three main diagnostic methods—palpation, vaginal ultrasound of the small pelvis, and determination of the concentration of CA-125 in the blood—lack sufficient sensitivity and specificity for this purpose. Each of these methods has its drawbacks: with manual palpation, the early stages of malignant tumors are indeterminate; with echography of the appendages, primary malignant ovarian tumors with infiltrative growth are not verified. Furthermore, while the accuracy of this method is 82.7%, the error in the diagnosis of ovarian cancer is 10–37% [59,60,61]. Errors depend on the degree of obesity of the abdominal wall, adhesions, fullness of the bladder, experience of the doctor, and solidification of the tumor structure [62].

Some advanced methods like CT for the in-depth diagnosis of malignant ovarian scan be used in cases where echography does not give a clear idea of the degree of tumor involvement. This method is costly and exposes the patient to a large radiation dose [63,64,65]. MRI is a more advanced method of radiation diagnostics for assessing the extent of the spread of a malignant tumor process than CT while exposing the patient to lower radiation exposure. However, the high cost of the equipment remains a limitation to its widespread use. Laparoscopic diagnostic methods provide significant assistance in the diagnosis of diseases of the pelvic organs. However, the techniques are invasive, difficult to use, and sometimes contraindicated in the adhesive process in the abdominal cavity of patients with severe somatic pathology [66]. A new method for diagnosing diseases of the pelvic organs is required. The use of MWR for this shows promise, although it has yet to be rigorously tested. Other methods such as internal temperature based on electromagnetic emission from human body could be used [67,68]

## 5. Conclusions

The search for strategies and diagnostic methods for the early detection of ovarian cancer remains an urgent public health problem owing to the high mortality rate from this disease. Current research is mainly focused on finding new biomarkers or developing multimodal algorithms that include both tumor markers and free DNA and ultrasound of the pelvic organs. Despite advances in the detection of ovarian cancer, there are still no convincing data from randomized controlled trials on the reduction of mortality rates with these new strategies. This has prevented the global adoption of these strategies for the screening and early diagnosis of ovarian cancer in national protocols [69]. 

The main principle for the correct examination of a patient is the observance of the algorithm of diagnostic measures: careful history taking, physical examination, ultrasound of the abdominal cavity (including of the small pelvis and retroperitoneal space), determination of the level of tumor markers in the blood serum, endometrial aspiration biopsy and microscopic study of the obtained material, and study of the karyotype (in patients with primary amenorrhea). The anamnesis of the disease allows one to determine the correct procedure. The interpretation of the patient’s complaints and physical and gynecological examinations make it possible to detect a potential malignant ovarian tumor and conduct an initial examination of the patient. Unfortunately, at the initial stages of the disease, epithelial tumors are asymptomatic. A high risk of malignant ovarian tumors is observed in women over the age of 49 with a burdened hereditary history (mutations of the BRCA1 and BRCA2 genes, Lynch syndrome, etc.) and after ovarian hyperstimulation, when using hormone replacement therapy, endometriosis, or obesity. Often, the semiotics of ovarian cancer is levelled by concomitant diseases. Furthermore, complaints in patients with ovarian cancer appear only when the tumor goes beyond the organ, which is why perimenopausal and elderly women with cardiological, gastroenterological, pulmonological, nephrological and, in particular, gynecological diseases should be referred to an oncologist for advice. Concerning surgical intervention, the important principles for suspected ovarian cancer are as follows: mandatory examination of the abdominal organs (including the small pelvis), taking a wash or ascitic fluid for cytological examination, removal of uterine appendages on the side of the affected ovary, a biopsy of the contralateral ovary, multiple biopsies of the peritoneum and all suspicious areas, urgent microscopic examination of the tumor, and conversion and radical surgery [70].

## Data Availability

Not applicable.

## References

[1] Urmancheeva A.F., Kutusheva G.F., Ulrikh E.A. (2012). Ovarian Tumors: Clinical Picture, Diagnosis and Treatment.

[2] Egunova M.A., Kutsenko I.G. (2016). Differential diagnosis of benign and malignant neoplasms of the ovaries (history of the issue). J. Obstet. Women’s Dis..

[3] Ionescu C.A., Matei A., Navolan D., Dimitriu M., Bohâltea R., Neacsu A., Ilinca C., Ples L. (2018). Correlation of ultrasound features and the Risk of Ovarian Malignancy Algorithm score for different histopathological subtypes of benign adnexal masses. Medicine.

[4] Kaprin A.D., Starinskiy V.V., Petrova G.V. (2018). The State of Cancer Care for the Population of RUSSIA.

[5] Hill K. (1996). The demography of menopause. Maturitas.

[6] Yureneva S.V., Ermakova E.I. (2017). The management of women with menopausal disorders (review of clinical guidelines). Probl. reproduktsii.

[7] Guraslan H., Dogan K. (2016). Management of unilocular or multilocular cysts more than 5 centimeters in postmenopausal women. Eur. J. Obstet. Gynecol. Reprod. Biol..

[8] (2017). Committee Opinion No. 716: The Role of the Obstetrician-Gynecologist in the Early Detection of Epithelial Ovarian Cancer in Women at Average Risk. Obstet. Gynecol..

[9] Alibakhshikenari M., Virdee B.S., Shukla P., Parchin N.O., Azpilicueta L., See C.H., Abd-Alhameed R.A., Falcone F., Huynen I., Denidni T.A. (2020). Metamaterial-Inspired Antenna Array for Application in Microwave Breast Imaging Systems for Tumor Detection. IEEE Access.

[10] Orr B., Edwards R.P. (2018). Diagnosis and Treatment of Ovarian Cancer. Hematol. Clin. N. Am..

[11] Vorobiev A.V., Protasova A.E. (2022). General questions of screening. J. Pract. Oncol..

[12] Urmancheeva A.F., Kutusheva G.F., Ulrikh E.A., Efimova O.A. (2017). Complex radiation diagnostics of ovarian tumor formations at the preoperative stage. Povolzhsky Oncol. Bull..

[13] Zola P., Macchi C., Cibula D., Colombo N., Kimmig R., Maggino T., Reed N., Kesic V. (2015). Follow-up in Gynecological Malignancies: A State of Art. Int. J. Gynecol. Cancer.

[14] (2018). Clinical Recommendations of the Ministry of Health of The Russian Federation “Diagnostics and Treatment of Benign Ovarian Neoplasms from the Perspective of Cancer Prevention”. http://zdrav.spb.ru.

[15] Stewart B.W., Wild C.P. (2014). World Cancer Report, 2014.

[16] Hammarström S. (1999). The carcinoembryonic antigen (CEA) family: Structures, suggested functions and expression in normal and malignant tissues. Semin. Cancer Biol..

[17] Khoo S.K., MacKay E.V. (1976). Carcinoembryonic antigen (CEA) in ovarian cancer: Factors influencing its incidence and changes which occur in response to cytotoxic drugs. BJOG Int. J. Obstet. Gynaecol..

[18] Bast R., Feeney M., Lazarus H., Nadler L.M., Colvin R.B., Knapp R.C. (1981). Reactivity of a monoclonal antibody with human ovarian carcinoma. J. Clin. Investig..

[19] Benjapibal M., Neungton C. (2007). Pre-operative prediction of serum CA125 level in women with ovarian masses. J. Med. Assoc. Thai..

[20] Wilbaux M., Hénin E., Oza A., Colomban O., Pujade-Lauraine E., Freyer G., Tod M., You B. (2014). Prediction of tumour response induced by chemotherapy using modelling of CA-125 kinetics in recurrent ovarian cancer patients. Br. J. Cancer.

[21] Li F., Tie R., Chang K., Wang F., Deng S., Lu W., Yu L., Chen M. (2012). Does risk for ovarian malignancy algorithm excel human epididymis protein 4 and ca125 in predicting epithelial ovarian cancer: A meta-analysis. BMC Cancer.

[22] Bian J., Li B., Kou X.J., Liu T.Z., Ming L. (2013). Clinical significance of combined detection of serum tumor markers in diagnosis of patients with ovarian cancer. Asian Pac. J. Cancer Prev..

[23] Robati M., Ghaderi A., Mehraban M., Shafizad A., Nasrolahi H., Mohammadianpanah M. (2013). Vascular endothelial growth factor (VEGF) improves the sensitivity of CA125 for differentiation of epithelial ovarian cancers from ovarian cysts. Arch. Gynecol. Obs..

[24] Havrilesky L.J., Whitehead C.M., Rubatt J.M., Cheek R.L., Groelke J., He Q., Malinowski D.P., Fischer T.J., Berchuck A. (2008). Evaluation of biomarker panels for early stage ovarian cancer detection and monitoring for disease recurrence. Gynecol. Oncol..

[25] Drapkin R., von Horsten H.H., Lin Y., Mok S.C., Crum C.P., Welch W.R., Hecht J.L. (2005). Human epididymis protein 4 (HE4) is a secreted glycoprotein that is overexpressed by serous and endometrioid ovarian carcinomas. Cancer Res..

[26] Lin J., Qin J., Sangvatanakul V. (2012). Human epididymis protein 4 for differential diagnosis between benign gynecologic disease and ovarian cancer: A systematic review and meta-analysis. Eur. J. Obstet. Gynecol. Reprod. Biol..

[27] Bast R.C., Skates S., Lokshin A., Moore R.G. (2012). Differential diagnosis of pelvic mass: Improved algorithms and novel biomarkers. Int. J. Gynecol. Cancer IGC.

[28] Zhen S., Bian L.H., Chang L.L., Gao X. (2014). Comparison of serum human epididymis protein 4 and carbohydrate antigen 125 as markers in ovarian cancer: A meta-analysis. Mol. Clin. Oncol..

[29] Wu C.M., Li X.L., Li L., Yin L.L., Li Q. (2014). Combined detection of tumor makers in the diagnosis of ovarian tumors. Int. J. Lab. Med..

[30] Jacobs I., Oram D., Fairbanks J., Turner J., Frost C., Grudzinskas J.G. (1990). A risk of malignancy index incorporating CA 125, ultrasound and menopausal status for the accurate preoperative diagnosis of ovarian cancer. Br. J. Obs. Gynaecol..

[31] Moore R.G., Jabre-Raughley M., Brown A.K., Robison K.M., Miller M.C., Allard W.J., Kurman R.J., Bast R.C., Skates S.J. (2010). Comparison of a novel multiple marker assay vs the Risk of Malignancy Index for the prediction of epithelial ovarian cancer in patients with a pelvic mass. Am. J. Obstet. Gynecol..

[32] Montagnana M., Danese E., Ruzzenente O., Bresciani V., Nuzzo T., Gelati M., Salvagno G., Franchi M., Lippi G., Guidi G. (2011). The ROMA (Risk of Ovarian Malignancy Algorithm) for estimating the risk of epithelial ovarian cancer in women presenting with pelvic mass: Is it really useful?. Clin. Chem. Lab. Med..

[33] Dayyani F., Uhlig S., Colson B., Simon K., Rolny V., Morgenstern D., Schlumbrecht M. (2016). Diagnostic performance of risk of ovarian malignancy algorithm against CA-125 and HE4 in connection with ovarian cancer: A meta-analysis. Int. J. Gynecol. Cancer.

[34] Kaijser J., Bourne T., Valentin L., Sayasneh A., Van Holsbeke C., Vergote I., Testa A.C., Franchi D., Van Calster B., Timmerman D. (2013). Improving strategies for diagnosing ovarian cancer: A summary of the International Ovarian Tumor Analysis (IOTA) studies. Ultrasound Obstet. Gynecol..

[35] Mehdi G., Maheshwari V., Afzal S., Ansari H.A., Ansari M. (2010). Image-guided fine-needle aspiration cytology of ovarian tumors: An assessment of diagnostic efficacy. J. Cytol..

[36] Dubrovskaya K.S. (2018). Diagnostics, Treatment and Prediction of the Outcomes of Pelvic Neoplasms in Gynecological Patients Abstract of the Dissertation for the Degree of Candidate of Medical Sciences.

[37] Borisova E.A. (2018). Comprehensive differential diagnosis of tumors of the uterine appendages. Abstract of the Dissertation of the Candidate of Medical Sciences.

[38] Suh-Burgmann E., Flanagan T., Osinski T., Alavi M., Herrinton L. (2018). Prospective Validation of a Standardized Ultrasonography-Based Ovarian Cancer Risk Assessment System. Obstet. Gynecol..

[39] Valentin L., Ameye L., Savelli L., Leone F.P.G., Czekierdowski A., Lissoni A.A., Fischerova D., Guerriero S., Van Holsbeke C., Van Huffel S. (2011). Adnexal masses difficult to classify as benign or malignant using subjective assessment of gray-scale and Doppler ultrasound findings: Logistic regression models do not help. Ultrasound Obstet. Gynecol..

[40] Meys E.M.J., Kaijser J., Kruitwagen R., Slangen B., Van Calster B., Aertgeerts B., Verbakel J., Timmerman D., Van Gorp T. (2016). Subjective assessment versus ultrasound models to diagnose ovarian cancer: A systematic review and meta-analysis. Eur. J. Cancer.

[41] Timmerman D., Planchamp F., Bourne T., Landolfo C., du Bois A., Chiva L., Cibula D., Concin N., Fischerova D., Froyman W. (2021). ESGO/ISUOG/IOTA/ESGE Consensus Statement on preoperative diagnosis of ovarian tumors. Ultrasound Obstet. Gynecol..

[42] Ngu S.F., Chai Y.K., Choi K.M., Leung T.W., Li J., Kwok G.S.T., Chu M.M.Y., Tse K.Y., Cheung V.Y.T., Ngan H.Y.S. (2022). Diagnostic Performance of Risk of Malignancy Algorithm (ROMA), Risk of Malignancy Index (RMI) and Expert Ultrasound Assessment in a Pelvic Mass Classified as Inconclusive by International Ovarian Tumour Analysis (IOTA) Simple Rules. Cancers.

[43] Carreras-Dieguez N., Glickman A., Munmany M., Casanovas G., Agustí N., Díaz-Feijoo B., Saco A., Sánchez B., Gaba L., Angeles M.A. (2022). Comparison of HE4, CA125, ROMA and CPH-I for Preoperative Assessment of Adnexal Tumors. Diagnostics.

[44] Sundar S., Rick C., Dowling F., Au P., Snell K., Rai N., Champaneria R., Stobart H., Neal R., Davenport C. (2016). Refining Ovarian Cancer Test accuracy Scores (ROCkeTS): Protocol for a prospective longitudinal test accuracy study to validate new risk scores in women with symptoms of suspected ovarian cancer. BMJ Open.

[45] Timmerman D., Ameye L., Fischerová D., Epstein E., Melis G.B., Guerriero S., Van Holsbeke C., Savelli L., Fruscio R., Lissoni A.A. (2010). Simple ultrasound rules to distinguish between benign and malignant adnexal masses before surgery: Prospective validation by IOTA group. BMJ.

[46] Froyman W., Landolfo C., De Cock B., Wynants L., Sladkevicius P., Testa A.C., Van Holsbeke C., Domali E., Fruscio R., Epstein E. (2019). Risk of complications in patients with conservatively managed ovarian tumors (IOTA5): A 2-year interim analysis of a multicenter, prospective, cohort study. Lancet Oncol..

[47] (2016). The Management of Ovarian Cysts in Postmenopausal Women, RCOG Greentop Guisline No. https://www-temp.rcog.org.uk/guidance/browse-all-guidance/green-top-guidelines/ovarian-cysts-in-postmenopausal-women-green-top-guideline-no-34/.

[48] (2016). Final Recommendation Statement: Ovarian Cancer: Screening. U.S. Preventive Services Task Force (USPSTF). https://www.uspreventiveservicestaskforce.org/Page/Document/RecommendationStatementFinal/ovarian-cancer-screening#:~:text=The%20USPSTF%20does%20not%20recommend,been%20evaluated%20in%20screening%20studies.

[49] Grossman D.C., Curry S.J., Owens D.K., Barry M.J., Davidson K.W., Doubeni C.A., Epling J.W., Kemper A.R., Krist A.H., Kurth A.E. (2018). Screening for Ovarian Cancer: US Preventive Services Task Force Recommendation Statement. US Preventive Services Task Force. JAMA.

[50] Davenport C., Rai N., Sharma P., Deeks J.J., Berhane S., Mallett S., Saha P., Champaneria R., Bayliss S.E., Snell K.I. (2022). Menopausal status, ultrasound and biomarker tests in combination for the diagnosis of ovarian cancer in symptomatic women. Cochrane Database Syst. Rev..

[51] Watrowski R., Obermayr E., Wallisch C., Aust S., Concin N., Braicu E.I., Van Gorp T., Hasenburg A., Sehouli J., Vergote I. (2022). Biomarker-Based Models for Preoper-ative Assessment of Adnexal Mass: A Multicenter Validation Study. Cancers.

[52] Funston G., Hamilton W., Abel G., Crosbie E.J., Rous B., Walter F.M. (2020). The diagnostic performance of CA125 for the detection of ovarian and non-ovarian cancer in primary care: A population-based cohort study. PLoS Med..

[53] Menon U., Gentry-Maharaj A., Burnell M., Singh N., Ryan A., Karpinskyj C., Carlino G., Taylor J., Massingham S.K., Raikou M. (2021). Ovarian cancer population screening and mortality after long-term follow-up in the UK Collaborative Trial of Ovarian Cancer Screening (UKCTOCS): A randomised controlled trial. Lancet.

[54] Gautherie M. (1982). Temperature and Blood Flow Patterns in Breast Cancer During Natural Evolution and Following Radiotherapy. Biomed. Thermol..

[55] Goryanin I., Karbainov S., Shevelev O., Tarakanov A., Redpath K., Vesnin S., Ivanov Y. (2020). Passive microwave radiometry in biomedical studies. Drug Discov. Today.

[56] Vesnin S., Turnbull A.K., Dixon J.M., Goryanin I. (2017). Modern microwave thermometry for breast cancer. J. Mol. Imaging Dyn..

[57] Li J., Galazis C., Popov L., Ovchinnikov L., Kharybina T., Vesnin S., Losev A., Goryanin I. (2022). Dynamic Weight Agnostic Neural Networks and Medical Microwave Radiometry (MWR) for Breast Cancer Diagnostics. Diagnostics.

[58] Mustafin C.K., Pak E.V. (2016). Method for Screening Diagnostics of Malignant Neoplasms of the Ovaries in Postmenopausal Women. Patent.

[59] (2013). National Comprehensive Cancer Network Clinical Practice Guidelines in Oncology. Ovarian Cancer.

[60] Starinsky V.V., Sergeeva N.S., Marshutina N.V., Korneeva I.A. (2013). Problems of early diagnosis and screening of ovarian cancer: Reality and prospects. Oncol. J. Them. PA.

[61] Siegel R., Naishadham D., Jemal A. (2012). Cancer Statistics, 2012. Cancer J. Clin..

[62] Rein B.J., Gupta S., Dada R., Safi J., Michener C., Agarwal A. (2011). Potential markers for detection and monitoring of ovarian cancer. J. Oncol..

[63] Sala E., Kataoka M., Pandit-Taskar N., Ishill N., Mironov S., Moskowitz C.S., Mironov O., Collins M.A., Chi D.S., Larson S. (2010). Recurrent ovarian cancer: Use of contrast-enhanced CT and PET/CT to accurately localize tumor recurrence and to predict patients’ survival. Radiology.

[64] Fernandes M.C., Nikolovski I., Long Roche K., Lakhman Y. (2022). CT of Ovarian Cancer for Primary Treatment Planning: What the Surgeon Needs to Know-Radiology In Training. Radiology.

[65] Feng S., Xia T., Ge Y., Zhang K., Ji X., Luo S., Shen Y. (2022). Computed Tomography Imaging-Based Radiogenomics Analysis Reveals Hypoxia Patterns and Immunological Characteristics in Ovarian Cancer. Front. Immunol..

[66] Van de Vrie R., Rutten M.J., Asseler J., Leeflang M.M.G., Kenter G.G., Mol B.J., Buist M. (2019). Laparoscopy for diagnosing resectability of disease in women with advanced ovarian cancer. Cochrane Database Syst. Rev..

[67] Makarov V.N., Shmeliova D.V., Boos N.A. (2021). Phantom to control the thermal ablation process. Russ. Technol. J..

[68] Kwon S., Lee S. (2016). Recent Advances in Microwave Imaging for Breast Cancer Detection. Int. J. Biomed. Imaging.

[69] Ferlay J., Shin H.R., Bray F. (2008). Globocan Cancer Incidence and Mortality Worldwide: IARC Cancer Base No. 10.

[70] Dodge J.E., Covens A., Lacchetti C., Elit L.M., Le T., Devries–Aboud M., Fung-Kee-Fung M. (2012). Management of a Suspicious Adnexal Mass: A Clinical Practice Guideline. Curr. Oncol..

